# (3,6-Dimeth­oxy­naphthalen-2-yl)(2,4,6-trimethyl­phen­yl)methanone

**DOI:** 10.1107/S1600536811042401

**Published:** 2011-10-29

**Authors:** Toyokazu Muto, Kosuke Sasagawa, Akiko Okamoto, Hideaki Oike, Noriyuki Yonezawa

**Affiliations:** a Department of Organic and Polymer Materials Chemistry, Tokyo University of Agriculture & Technology, 2-24-16 Naka-machi, Koganei, Tokyo 184-8588, Japan

## Abstract

In the title compound, C_22_H_22_O_3_, the dihedral angle between the naphthalene ring system and the benzene ring is 79.95 (5)°. The bridging carbonyl C—C(=O)—C group makes dihedral angles of 24.21 (7) and 82.43 (8)°, respectively, with the naphthalene ring system and the benzene ring. In the crystal, weak inter­molecular C—H⋯O inter­actions link mol­ecules into chains parallel to the *c* axis.

## Related literature

For electrophilic aromatic substitution of naphthalene derivatives, see: Okamoto & Yonezawa (2009 ▶); Okamoto *et al.* (2011 ▶). For the structures of closely related compounds, see: Muto *et al.* (2010 ▶, 2011 ▶); Kato *et al.* (2010 ▶, 2011 ▶).
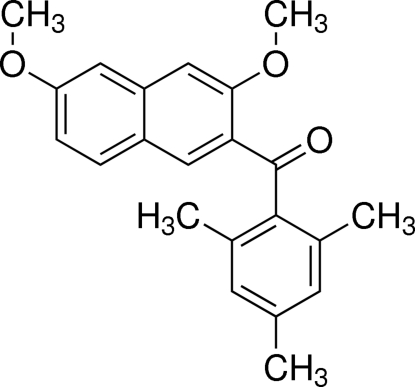


## Experimental

### 

#### Crystal data

C_22_H_22_O_3_
*M* *_r_* = 334.40
Monoclinic, 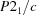

*a* = 15.4205 (2) Å
*b* = 8.23702 (10) Å
*c* = 15.3832 (2) Å
β = 111.30 (1)°
*V* = 1820.49 (13) Å^3^
*Z* = 4
Cu *K*α radiation
μ = 0.64 mm^−1^
*T* = 193 K
0.60 × 0.20 × 0.10 mm

#### Data collection

Rigaku R-AXIS RAPID diffractometer
Absorption correction: numerical (*NUMABS*; Higashi 1999 ▶) *T*_min_ = 0.701, *T*_max_ = 0.939
32532 measured reflections
3310 independent reflections
3047 reflections with *I* > 2σ(*I*)
*R*_int_ = 0.035

#### Refinement

*R*[*F* ^2^ > 2σ(*F* ^2^)] = 0.043
*wR*(*F* ^2^) = 0.130
*S* = 1.04
3310 reflections
232 parameters
H-atom parameters constrained
Δρ_max_ = 0.24 e Å^−3^
Δρ_min_ = −0.21 e Å^−3^

### 

Data collection: *PROCESS-AUTO* (Rigaku, 1998 ▶); cell refinement: *PROCESS-AUTO*; data reduction: *CrystalStructure* (Rigaku/MSC, 2004 ▶); program(s) used to solve structure: *SIR2004* (Burla *et al.*, 2005 ▶); program(s) used to refine structure: *SHELXL97* (Sheldrick, 2008 ▶); molecular graphics: *ORTEPIII* (Burnett & Johnson, 1996 ▶); software used to prepare material for publication: *SHELXL97*.

## Supplementary Material

Crystal structure: contains datablock(s) I, global. DOI: 10.1107/S1600536811042401/rz2647sup1.cif

Structure factors: contains datablock(s) I. DOI: 10.1107/S1600536811042401/rz2647Isup2.hkl

Supplementary material file. DOI: 10.1107/S1600536811042401/rz2647Isup3.cml

Additional supplementary materials:  crystallographic information; 3D view; checkCIF report

## Figures and Tables

**Table 1:** Hydrogen-bond geometry (Å, °)

*D*—H⋯*A*	*D*—H	H⋯*A*	*D*⋯*A*	*D*—H⋯*A*
C6—H6⋯O1^i^	0.95	2.30	3.1123 (19)	143

Symmetry code: (i) 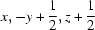.

**Fig. 1.**
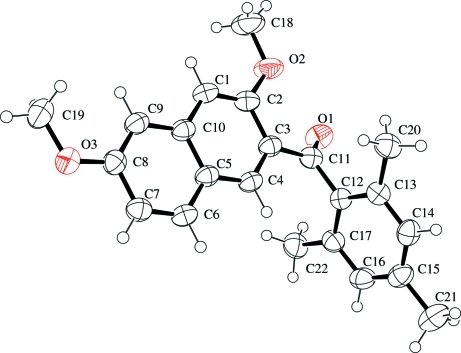
The molecular structure of the title compound with displacement ellipsoids drawn at the 50% probability level.

**Fig. 2.**
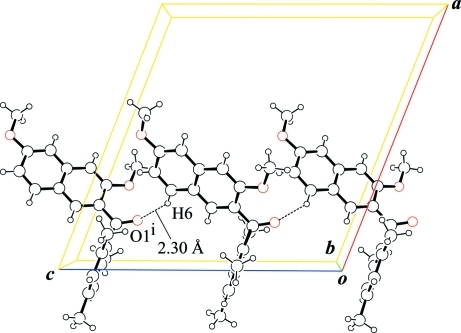
Intermolecular C6—H6···O1i interactions, viewed along the b axis [symmetry code: (i) x, -y + 1/2, z + 1/2].

## supplementary crystallographic information

### Comment

In the course of our study on electrophilic aromatic aroylation of 2,7-dimethoxynaphthalene, *peri*-aroylnaphthalene compounds have proven to be formed regioselectively with the aid of suitable acidic mediators (Okamoto & Yonezawa, 2009; Okamoto *et al.*, 2011). Recently, we have reported the crystal structures of several 1,8-diaroylated naphthalene analogues exemplified by 1,8-bis(4-methylbenzoyl)-2,7-dimethoxynaphthalene (Muto *et al.*, 2010). The aroyl groups at the 1,8-positions of the naphthalene rings in these compounds are connected in an almost perpendicular fashion. Besides, the crystal structures of 1-monoaroylated naphthalene derivatives and the *β*-isomers of 3-monoaroylated naphthalene derivatives have been also clarified such as (2,7-dimethoxynaphthalen-1-yl)(2,4,6-trimethylphenyl)methanone (Muto *et al.*, 2011), (3,6-dimethoxynaphthalen-2-yl)(phenyl)methanone (Kato *et al.*, 2011) and (4-bromophenyl)(3,6-dimethoxy-2-naphthyl)methanone (Kato *et al.*, 2010). As a part of our continuing study on the molecular structures of these homologous molecules, the crystal structure of title compound, 3-monoaroylnaphthalene bearing three methyl groups, is discussed in this report.

The molecular structure of the title compound is displayed in Fig. 1. The 2,4,6-trimethylphenyl group is situated out of the plane of the naphthalene ring. The dihedral angle between the best planes of the 2,4,6-trimethylphenyl ring (C12—C17) and the naphthalene ring (C1—C10) is 79.95 (5)°. The carbonyl moiety attaches almost coplanarly to the naphthalene ring rather than the benzene ring. The bridging carbonyl C—C(═O)—C plane makes dihedral angles of 24.21 (7) and 82.43 (8)°, respectively, with the naphthalene ring system and the benzene ring. The dihedral angle of the title compound between the bridging carbonyl plane and the naphthalene ring is smaller than those in 3-monoaroylated naphthalene analogues, (3,6-dimethoxynaphthalen-2-yl)(phenyl)methanone (Kato *et al.*, 2011) and (4-bromophenyl)(3,6-dimethoxy-2-naphthyl)methanone [54.32 (5) and 47.07 (9)°, respectively]. The carbonyl group makes torsion angles of 21.8 (2) and -98.12 (16)°, respectively, with the naphthalene ring and the benzene ring [C2—C3—C11—O1 torsion angle = 21.8 (2)°; O1—C11—C12—C13 torsion angle = -98.12 (16)°].

In the crystal structure, the molecular packing of the title compound is stabilized mainly by van der Waals interactions. The crystal packing is additionally stabilized by intermolecular C—H···O hydrogen bonding between the oxygen atom (O1) of the carbonyl group and one hydrogen atom (H6) of the naphthalene ring of the adjacent molecule along the *c* axis (Fig. 2; Table 1).

### Experimental

To a 100 ml flask, 2,4,6-trimethylbenzoyl chloride (30.0 mmol, 5.48 g), titanium chloride (90.0 mmol, 17.1 g) and methylene chloride (25 ml) were placed and stirred at rt. To the reaction mixture thus obtained, 2,7-dimethoxynaphthalene (10.0 mmol, 1.88 g) was added. After the reaction mixture was stirred at rt for 24 h, it was poured into ice-cold water (50 ml). The aqueous layer was extracted with CHCl_3_ (20 ml × 3). The combined extracts were washed with 2 *M* aqueous NaOH followed by washing with brine. The organic layers thus obtained were dried over anhydrous MgSO_4_. The solvent was removed under reduced pressure to give cake. The crude product was purified by recrystallization from hexane and CHCl_3_ (yield 56%). Colourless platelet single crystals suitable for X-ray diffraction were obtained by repeated crystallization from hexane/CHCl_3_ mixtures (3:1 *v*/*v*).

^1^H NMR δ (300 MHz, CDCl_3_); 2.14 (6*H*, s), 2.33 (3*H*, s), 3.92 (6*H*, s), 6.87 (2*H*, s), 6.98 (1*H*, dd, *J* = 2.4, 8.7 Hz), 7.04 (1*H*, d, *J* = 2.4 Hz), 7.12 (1*H*, s), 7.61 (1*H*, d, *J* = 9.0 Hz), 7.90 (1*H*, s) ppm.

^13^C NMR δ (75 MHz, CDCl_3_); 19.50, 21.12, 55.31, 55.82, 104.68, 106.17, 117.21, 122.93, 126.94, 128.31, 130.81, 134.06, 134.23, 138.10, 138.51, 139.18, 157.13, 160.25, 199.32 ppm.

IR (KBr); 1673 (C=O), 1626, 1499, 1470 (Ar, naphthalene), 1212 (=C—O—C) cm^-1^

HRMS (*m/z*); [*M* + H]^+^ Calcd for C_22_H_23_O_3_, 335.1647; found, 335.1640.

m.p. = 423.0–424.5 K

### Refinement

All H atoms were found in a difference map and were subsequently refined as riding atoms, with C—H = 0.95 (aromatic) and 0.98 (methyl) Å, and with *U*_ĩso_(H) = 1.2 *U*_eq_(C).

### Crystal data

**Table:**

C_22_H_22_O_3_	*F*(000) = 712
*M**_r_* = 334.40	*D*_x_ = 1.220 Mg m^−^^3^
Monoclinic, *P*2_1_/*c*	Cu *K*α radiation, λ = 1.54187 Å
Hall symbol: -P 2ybc	Cell parameters from 29183 reflections
*a* = 15.4205 (2) Å	θ = 3.1–68.3°
*b* = 8.23702 (10) Å	µ = 0.64 mm^−^^1^
*c* = 15.3832 (2) Å	*T* = 193 K
β = 111.30 (1)°	Platelet, colourless
*V* = 1820.49 (13) Å^3^	0.60 × 0.20 × 0.10 mm
*Z* = 4	

### Data collection

**Table:**

Rigaku R-AXIS RAPID diffractometer	3310 independent reflections
Radiation source: rotating anode	3047 reflections with *I* > 2σ(*I*)
graphite	*R*_int_ = 0.035
Detector resolution: 10.000 pixels mm^-1^	θ_max_ = 68.2°, θ_min_ = 3.1°
ω scans	*h* = −18→18
Absorption correction: numerical (*NUMABS*; Higashi 1999)	*k* = −9→9
*T*_min_ = 0.701, *T*_max_ = 0.939	*l* = −18→18
32532 measured reflections	

### Refinement

**Table:**

Refinement on *F*^2^	Secondary atom site location: difference Fourier map
Least-squares matrix: full	Hydrogen site location: inferred from neighbouring sites
*R*[*F*^2^ > 2σ(*F*^2^)] = 0.043	H-atom parameters constrained
*wR*(*F*^2^) = 0.130	*w* = 1/[σ^2^(*F*_o_^2^) + (0.0753*P*)^2^ + 0.4568*P*] where *P* = (*F*_o_^2^ + 2*F*_c_^2^)/3
*S* = 1.04	(Δ/σ)_max_ < 0.001
3310 reflections	Δρ_max_ = 0.24 e Å^−^^3^
232 parameters	Δρ_min_ = −0.21 e Å^−^^3^
0 restraints	Extinction correction: *SHELXL97* (Sheldrick, 2008), Fc^*^=kFc[1+0.001xFc^2^λ^3^/sin(2θ)]^-1/4^
Primary atom site location: structure-invariant direct methods	Extinction coefficient: 0.0049 (5)

### Special details

**Table:**

Geometry. All e.s.d.'s (except the e.s.d. in the dihedral angle between two l.s. planes) are estimated using the full covariance matrix. The cell e.s.d.'s are taken into account individually in the estimation of e.s.d.'s in distances, angles and torsion angles; correlations between e.s.d.'s in cell parameters are only used when they are defined by crystal symmetry. An approximate (isotropic) treatment of cell e.s.d.'s is used for estimating e.s.d.'s involving l.s. planes.
Refinement. Refinement of *F*^2^ against ALL reflections. The weighted *R*-factor *wR* and goodness of fit *S* are based on *F*^2^, conventional *R*-factors *R* are based on *F*, with *F* set to zero for negative *F*^2^. The threshold expression of *F*^2^ > σ(*F*^2^) is used only for calculating *R*-factors(gt) *etc*. and is not relevant to the choice of reflections for refinement. *R*-factors based on *F*^2^ are statistically about twice as large as those based on *F*, and *R*- factors based on ALL data will be even larger.

### Fractional atomic coordinates and isotropic or equivalent isotropic displacement parameters (Å^2^)

**Table:**

	*x*	*y*	*z*	*U*_iso_*/*U*_eq_	
O1	0.17897 (7)	0.11520 (14)	0.30253 (7)	0.0547 (3)	
O2	0.31350 (9)	−0.09944 (15)	0.39268 (8)	0.0644 (4)	
O3	0.51070 (7)	0.05027 (14)	0.89570 (7)	0.0524 (3)	
C1	0.38282 (10)	−0.06216 (18)	0.55932 (10)	0.0469 (4)	
H1	0.4328	−0.1328	0.5633	0.056*	
C2	0.31489 (10)	−0.03276 (18)	0.47401 (10)	0.0463 (3)	
C3	0.23765 (9)	0.07077 (17)	0.46641 (9)	0.0407 (3)	
C4	0.23345 (9)	0.13606 (16)	0.54713 (9)	0.0399 (3)	
H4	0.1812	0.2009	0.5431	0.048*	
C5	0.30303 (9)	0.11102 (16)	0.63517 (10)	0.0397 (3)	
C6	0.29974 (9)	0.18543 (18)	0.71684 (10)	0.0443 (3)	
H6	0.2481	0.2519	0.7130	0.053*	
C7	0.36953 (9)	0.16311 (18)	0.80084 (10)	0.0456 (3)	
H7	0.3667	0.2145	0.8550	0.055*	
C8	0.44640 (9)	0.06316 (17)	0.80730 (10)	0.0424 (3)	
C9	0.45182 (9)	−0.01199 (17)	0.73013 (10)	0.0433 (3)	
H9	0.5036	−0.0793	0.7357	0.052*	
C10	0.38003 (9)	0.01067 (16)	0.64181 (10)	0.0406 (3)	
C11	0.16556 (9)	0.11889 (16)	0.37545 (9)	0.0407 (3)	
C12	0.07397 (9)	0.18112 (16)	0.37644 (8)	0.0380 (3)	
C13	0.00479 (9)	0.07062 (17)	0.37435 (9)	0.0420 (3)	
C14	−0.08108 (10)	0.1316 (2)	0.37005 (10)	0.0475 (4)	
H14	−0.1293	0.0576	0.3672	0.057*	
C15	−0.09821 (10)	0.2967 (2)	0.36976 (10)	0.0500 (4)	
C16	−0.02745 (11)	0.40317 (18)	0.37347 (10)	0.0503 (4)	
H16	−0.0381	0.5166	0.3743	0.060*	
C17	0.05866 (10)	0.34880 (17)	0.37602 (9)	0.0435 (3)	
C18	0.38758 (15)	−0.2040 (3)	0.39608 (14)	0.0781 (6)	
H18A	0.3878	−0.2989	0.4346	0.094*	
H18B	0.4468	−0.1460	0.4232	0.094*	
H18C	0.3793	−0.2396	0.3328	0.094*	
C19	0.58995 (11)	−0.0495 (2)	0.90841 (12)	0.0635 (5)	
H19A	0.6244	−0.0067	0.8710	0.076*	
H19B	0.5696	−0.1606	0.8885	0.076*	
H19C	0.6304	−0.0496	0.9744	0.076*	
C20	0.02088 (11)	−0.10973 (18)	0.37508 (11)	0.0529 (4)	
H20A	0.0609	−0.1438	0.4379	0.063*	
H20B	0.0511	−0.1362	0.3308	0.063*	
H20C	−0.0389	−0.1665	0.3571	0.063*	
C21	−0.19204 (12)	0.3592 (3)	0.36448 (14)	0.0696 (5)	
H21A	−0.2273	0.2709	0.3788	0.084*	
H21B	−0.2263	0.4003	0.3015	0.084*	
H21C	−0.1834	0.4472	0.4098	0.084*	
C22	0.13333 (12)	0.46853 (19)	0.37718 (12)	0.0572 (4)	
H22A	0.1914	0.4422	0.4287	0.069*	
H22B	0.1136	0.5786	0.3857	0.069*	
H22C	0.1435	0.4624	0.3180	0.069*	

### Atomic displacement parameters (Å^2^)

**Table:**

	*U*^11^	*U*^22^	*U*^33^	*U*^12^	*U*^13^	*U*^23^
O1	0.0597 (6)	0.0639 (7)	0.0500 (6)	0.0070 (5)	0.0312 (5)	0.0096 (5)
O2	0.0725 (7)	0.0710 (8)	0.0509 (6)	0.0287 (6)	0.0238 (5)	−0.0071 (5)
O3	0.0453 (6)	0.0567 (7)	0.0503 (6)	0.0041 (4)	0.0116 (4)	−0.0015 (5)
C1	0.0439 (7)	0.0456 (8)	0.0553 (8)	0.0112 (6)	0.0228 (6)	−0.0012 (6)
C2	0.0508 (8)	0.0445 (8)	0.0490 (8)	0.0064 (6)	0.0247 (6)	−0.0028 (6)
C3	0.0409 (7)	0.0388 (7)	0.0467 (7)	0.0020 (5)	0.0209 (6)	0.0024 (5)
C4	0.0377 (6)	0.0392 (7)	0.0484 (7)	0.0044 (5)	0.0222 (6)	0.0031 (5)
C5	0.0378 (7)	0.0380 (7)	0.0485 (7)	0.0014 (5)	0.0221 (6)	0.0019 (5)
C6	0.0426 (7)	0.0456 (8)	0.0503 (8)	0.0071 (6)	0.0236 (6)	0.0013 (6)
C7	0.0475 (7)	0.0465 (8)	0.0475 (7)	0.0013 (6)	0.0228 (6)	−0.0019 (6)
C8	0.0385 (7)	0.0409 (7)	0.0475 (7)	−0.0038 (5)	0.0154 (6)	0.0022 (6)
C9	0.0366 (7)	0.0402 (7)	0.0554 (8)	0.0038 (5)	0.0195 (6)	0.0019 (6)
C10	0.0386 (7)	0.0374 (7)	0.0503 (7)	0.0012 (5)	0.0215 (6)	0.0019 (6)
C11	0.0457 (7)	0.0356 (7)	0.0455 (7)	−0.0027 (5)	0.0220 (6)	0.0023 (5)
C12	0.0405 (6)	0.0388 (7)	0.0353 (6)	0.0013 (5)	0.0145 (5)	0.0024 (5)
C13	0.0472 (7)	0.0414 (7)	0.0396 (7)	−0.0021 (6)	0.0185 (6)	0.0016 (5)
C14	0.0426 (7)	0.0572 (9)	0.0459 (7)	−0.0040 (6)	0.0199 (6)	0.0028 (6)
C15	0.0478 (8)	0.0611 (9)	0.0431 (7)	0.0109 (7)	0.0188 (6)	0.0053 (6)
C16	0.0560 (9)	0.0426 (8)	0.0511 (8)	0.0118 (6)	0.0181 (7)	0.0028 (6)
C17	0.0483 (8)	0.0382 (7)	0.0417 (7)	0.0012 (6)	0.0138 (6)	0.0021 (5)
C18	0.0894 (13)	0.0862 (14)	0.0635 (11)	0.0390 (11)	0.0334 (10)	−0.0077 (10)
C19	0.0438 (8)	0.0745 (12)	0.0640 (10)	0.0091 (8)	0.0098 (7)	0.0015 (8)
C20	0.0618 (9)	0.0403 (8)	0.0610 (9)	−0.0061 (7)	0.0276 (8)	0.0017 (7)
C21	0.0572 (10)	0.0882 (13)	0.0698 (11)	0.0210 (9)	0.0307 (8)	0.0101 (10)
C22	0.0605 (9)	0.0403 (8)	0.0660 (10)	−0.0050 (7)	0.0172 (8)	0.0037 (7)

### Geometric parameters (Å, °)

**Table:**

O1—C11	1.2126 (16)	C13—C14	1.395 (2)
O2—C2	1.3595 (17)	C13—C20	1.505 (2)
O2—C18	1.4166 (19)	C14—C15	1.385 (2)
O3—C8	1.3653 (17)	C14—H14	0.9500
O3—C19	1.4258 (19)	C15—C16	1.385 (2)
C1—C2	1.371 (2)	C15—C21	1.510 (2)
C1—C10	1.418 (2)	C16—C17	1.388 (2)
C1—H1	0.9500	C16—H16	0.9500
C2—C3	1.4347 (19)	C17—C22	1.511 (2)
C3—C4	1.3761 (19)	C18—H18A	0.9800
C3—C11	1.4903 (19)	C18—H18B	0.9800
C4—C5	1.404 (2)	C18—H18C	0.9800
C4—H4	0.9500	C19—H19A	0.9800
C5—C6	1.4153 (19)	C19—H19B	0.9800
C5—C10	1.4196 (18)	C19—H19C	0.9800
C6—C7	1.361 (2)	C20—H20A	0.9800
C6—H6	0.9500	C20—H20B	0.9800
C7—C8	1.4167 (19)	C20—H20C	0.9800
C7—H7	0.9500	C21—H21A	0.9800
C8—C9	1.368 (2)	C21—H21B	0.9800
C9—C10	1.419 (2)	C21—H21C	0.9800
C9—H9	0.9500	C22—H22A	0.9800
C11—C12	1.5077 (18)	C22—H22B	0.9800
C12—C13	1.3938 (19)	C22—H22C	0.9800
C12—C17	1.401 (2)		
			
C2—O2—C18	117.96 (13)	C15—C14—H14	119.0
C8—O3—C19	117.24 (12)	C13—C14—H14	119.0
C2—C1—C10	121.38 (12)	C16—C15—C14	118.37 (13)
C2—C1—H1	119.3	C16—C15—C21	120.77 (15)
C10—C1—H1	119.3	C14—C15—C21	120.86 (15)
O2—C2—C1	124.10 (13)	C15—C16—C17	121.88 (14)
O2—C2—C3	115.47 (12)	C15—C16—H16	119.1
C1—C2—C3	120.41 (12)	C17—C16—H16	119.1
C4—C3—C2	117.94 (12)	C16—C17—C12	118.43 (13)
C4—C3—C11	118.67 (12)	C16—C17—C22	120.44 (14)
C2—C3—C11	123.27 (12)	C12—C17—C22	121.13 (13)
C3—C4—C5	122.88 (12)	O2—C18—H18A	109.5
C3—C4—H4	118.6	O2—C18—H18B	109.5
C5—C4—H4	118.6	H18A—C18—H18B	109.5
C4—C5—C6	122.09 (12)	O2—C18—H18C	109.5
C4—C5—C10	118.74 (12)	H18A—C18—H18C	109.5
C6—C5—C10	119.15 (12)	H18B—C18—H18C	109.5
C7—C6—C5	120.89 (12)	O3—C19—H19A	109.5
C7—C6—H6	119.6	O3—C19—H19B	109.5
C5—C6—H6	119.6	H19A—C19—H19B	109.5
C6—C7—C8	119.88 (13)	O3—C19—H19C	109.5
C6—C7—H7	120.1	H19A—C19—H19C	109.5
C8—C7—H7	120.1	H19B—C19—H19C	109.5
O3—C8—C9	125.30 (12)	C13—C20—H20A	109.5
O3—C8—C7	113.68 (12)	C13—C20—H20B	109.5
C9—C8—C7	121.02 (13)	H20A—C20—H20B	109.5
C8—C9—C10	119.93 (12)	C13—C20—H20C	109.5
C8—C9—H9	120.0	H20A—C20—H20C	109.5
C10—C9—H9	120.0	H20B—C20—H20C	109.5
C1—C10—C9	122.29 (12)	C15—C21—H21A	109.5
C1—C10—C5	118.58 (13)	C15—C21—H21B	109.5
C9—C10—C5	119.12 (12)	H21A—C21—H21B	109.5
O1—C11—C3	122.83 (12)	C15—C21—H21C	109.5
O1—C11—C12	119.54 (12)	H21A—C21—H21C	109.5
C3—C11—C12	117.58 (11)	H21B—C21—H21C	109.5
C13—C12—C17	121.16 (13)	C17—C22—H22A	109.5
C13—C12—C11	119.32 (12)	C17—C22—H22B	109.5
C17—C12—C11	119.48 (12)	H22A—C22—H22B	109.5
C12—C13—C14	118.13 (13)	C17—C22—H22C	109.5
C12—C13—C20	121.43 (13)	H22A—C22—H22C	109.5
C14—C13—C20	120.43 (13)	H22B—C22—H22C	109.5
C15—C14—C13	122.01 (14)		
			
C18—O2—C2—C1	−0.9 (3)	C4—C5—C10—C9	178.42 (12)
C18—O2—C2—C3	−179.40 (16)	C6—C5—C10—C9	−0.18 (19)
C10—C1—C2—O2	−179.68 (14)	C4—C3—C11—O1	154.26 (14)
C10—C1—C2—C3	−1.3 (2)	C2—C3—C11—O1	−21.7 (2)
O2—C2—C3—C4	177.30 (13)	C4—C3—C11—C12	−23.10 (18)
C1—C2—C3—C4	−1.2 (2)	C2—C3—C11—C12	160.89 (13)
O2—C2—C3—C11	−6.7 (2)	O1—C11—C12—C13	98.12 (16)
C1—C2—C3—C11	174.82 (13)	C3—C11—C12—C13	−84.42 (15)
C2—C3—C4—C5	2.7 (2)	O1—C11—C12—C17	−79.58 (17)
C11—C3—C4—C5	−173.52 (12)	C3—C11—C12—C17	97.88 (15)
C3—C4—C5—C6	176.92 (13)	C17—C12—C13—C14	1.18 (19)
C3—C4—C5—C10	−1.6 (2)	C11—C12—C13—C14	−176.48 (12)
C4—C5—C6—C7	−177.94 (13)	C17—C12—C13—C20	−179.81 (13)
C10—C5—C6—C7	0.6 (2)	C11—C12—C13—C20	2.53 (19)
C5—C6—C7—C8	−0.5 (2)	C12—C13—C14—C15	−1.3 (2)
C19—O3—C8—C9	0.1 (2)	C20—C13—C14—C15	179.64 (14)
C19—O3—C8—C7	179.45 (13)	C13—C14—C15—C16	0.3 (2)
C6—C7—C8—O3	−179.41 (13)	C13—C14—C15—C21	179.64 (13)
C6—C7—C8—C9	0.0 (2)	C14—C15—C16—C17	1.0 (2)
O3—C8—C9—C10	179.76 (12)	C21—C15—C16—C17	−178.38 (14)
C7—C8—C9—C10	0.5 (2)	C15—C16—C17—C12	−1.1 (2)
C2—C1—C10—C9	−176.96 (14)	C15—C16—C17—C22	178.19 (13)
C2—C1—C10—C5	2.4 (2)	C13—C12—C17—C16	0.0 (2)
C8—C9—C10—C1	178.97 (13)	C11—C12—C17—C16	177.66 (12)
C8—C9—C10—C5	−0.3 (2)	C13—C12—C17—C22	−179.30 (12)
C4—C5—C10—C1	−0.91 (19)	C11—C12—C17—C22	−1.64 (19)
C6—C5—C10—C1	−179.52 (13)		

### Hydrogen-bond geometry (Å, °)

**Table:**

*D*—H···*A*	*D*—H	H···*A*	*D*···*A*	*D*—H···*A*
C6—H6···O1^i^	0.95	2.30	3.1123 (19)	143

Symmetry codes: (i) *x*, −*y*+1/2, *z*+1/2.

## References

[bb1] Burla, M. C., Caliandro, R., Camalli, M., Carrozzini, B., Cascarano, G. L., De Caro, L., Giacovazzo, C., Polidori, G. & Spagna, R. (2005). *J. Appl. Cryst.* **38**, 381–388.

[bb2] Burnett, M. N. & Johnson, C. K. (1996). *ORTEPIII.* Report ORNL-6895. Oak Ridge National Laboratory, Tennessee, USA.

[bb3] Higashi, T. (1999). *NUMABS.* Rigaku Corporation, Tokyo, Japan.

[bb4] Kato, Y., Nagasawa, A., Kataoka, K., Okamoto, A. & Yonezawa, N. (2010). *Acta Cryst.* E**66**, o2795. 10.1107/S160053681004016X PMC3009183 21588992

[bb5] Kato, Y., Takeuchi, R., Muto, T., Okamoto, A. & Yonezawa, N. (2011). *Acta Cryst.* E**67**, o668. 10.1107/S1600536811005630 PMC3052025 21522417

[bb6] Muto, T., Kato, Y., Nagasawa, A., Okamoto, A. & Yonezawa, N. (2010). *Acta Cryst.* E**66**, o2752. 10.1107/S1600536810039620 PMC3009178 21588956

[bb7] Muto, T., Sasagawa, K., Okamoto, A., Oike, H. & Yonezawa, N. (2011). *Acta Cryst.* E**67**, o2813. 10.1107/S1600536811039225 PMC3201449 22064621

[bb8] Okamoto, A., Mitsui, R., Oike, H. & Yonezawa, N. (2011). *Chem. Lett.* **40**, 1283–1284.

[bb9] Okamoto, A. & Yonezawa, N. (2009). *Chem. Lett.* **38**, 914–915.

[bb10] Rigaku (1998). *PROCESS-AUTO.* Rigaku Corporation, Tokyo, Japan.

[bb11] Rigaku/MSC (2004). *CrystalStructure.* Rigaku/MSC, The Woodlands, Texas, USA.

[bb12] Sheldrick, G. M. (2008). *Acta Cryst.* A**64**, 112–122. 10.1107/S0108767307043930 18156677

